# Ultra‐Flat Watt‐Level All‐Fiber Supercontinuum Generation With 3‐dB Spectral Bandwidth Spanning From 414 to 1860 nm

**DOI:** 10.1002/nap2.70058

**Published:** 2026-03-28

**Authors:** Yashuai Guo, Xiaohong Hu, Ting Zhang, Ran Pan, Wei Zhang, Yishan Wang, Wei Zhao

**Affiliations:** ^1^ State Key Laboratory of Ultrafast Optical Science and Technology Xi'an Institute of Optics and Precision Mechanics Chinese Academy of Sciences Xi'an China; ^2^ University of Chinese Academy of Sciences Beijing China

**Keywords:** fiber laser, photonic crystal fiber, ultra‐flat supercontinuum, visible light band

## Abstract

We propose a method for generating ultraflat supercontinuum (SC) spanning from the visible to near‐infrared (NIR) region. Specifically, through both the numerical simulations and experiments, we confirm that an optimized combination of pump pulse chirp, peak power, and dispersion of highly nonlinear photonic crystal fiber (PCF) can effectively enhance the four‐wave mixing (FWM) effect which is dominant for the enhancement of visible spectral components and the extending of the short‐wavelength boundary. In the experiment, we produce 4.35‐W ultraflat SC covering 414–1860 nm (3‐dB bandwidth and 2.12‐octave) which encompasses nearly the entire visible band. To the best of our knowledge, this work reports the broadest visible to NIR ultraflat (3‐dB bandwidth) SC source ever reported, which also exhibits the maximum visible band coverage. The proposed approach holds significant application potential for fields in which visible band coverage and SC flatness are extremely demanded, such as remote sensing detection.

## Graphical abstract

We experimentally achieve an ultraflat, 4.35‐W all‐fiber supercontinuum spanning from 414 to 1860 nm (3‐dB bandwidth). To our knowledge, this represents the broadest octave‐spanning, visible‐to‐NIR, ultraflat SC source ever reported, which also exhibits maximal visible band coverage.

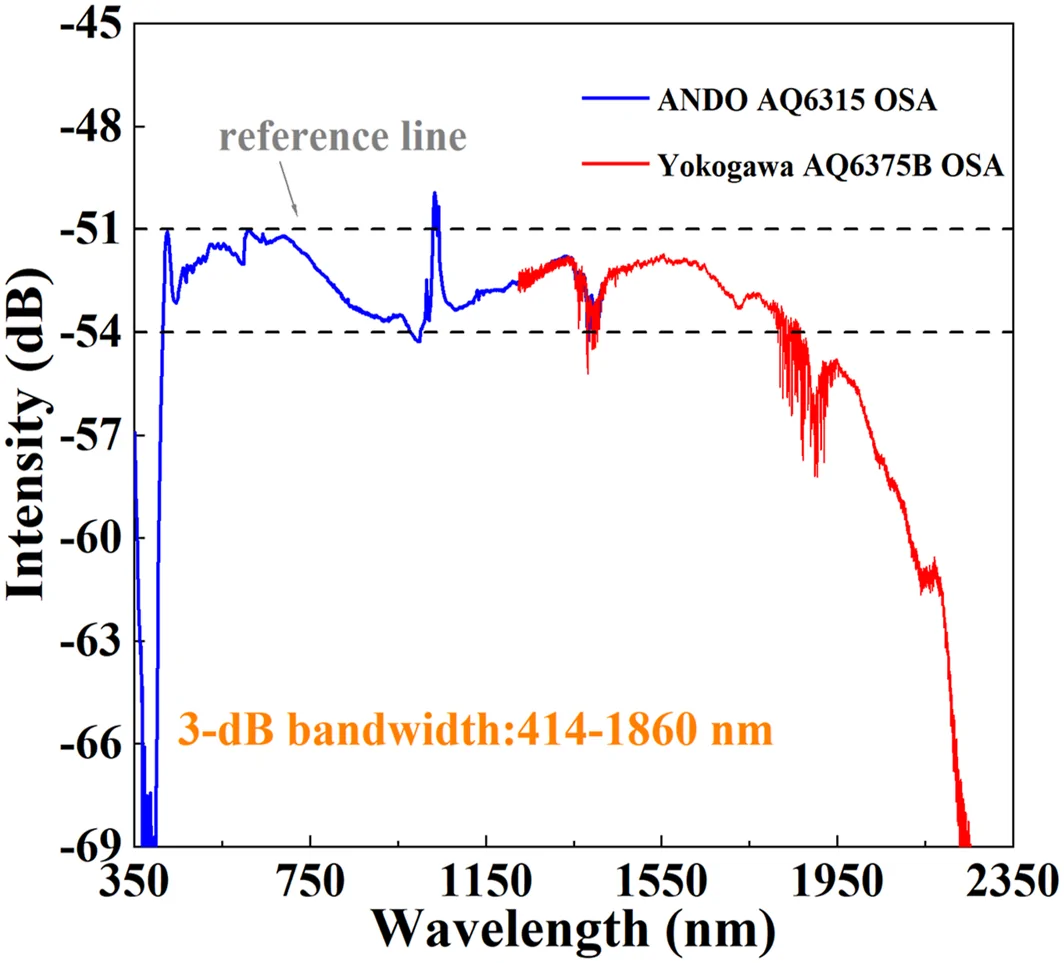

## 1 Introduction

Supercontinuum (SC) generation by injecting high‐peak‐power pulsed light, which is delivered by a master oscillator power amplifier (MOPA) system, into highly nonlinear photonic crystal fiber (PCF) has been studied for decades. This approach benefits from the high nonlinearity and flexible dispersion tunability of PCF. Significant progresses have been achieved in almost all aspects of SC, including the average power, spectral coverage, flatness, polarization state, and beam quality [1, 2, 3, 4, 5, 6, 7]. Due to its broad spectrum, high coherence, and superior beam quality, combined with compactness and high reliability of fiber lasers, SC sources garner significant favor in both scientific and industrial communities. As demonstrated in the following sections, SC sources covering the visible to near‐infrared (NIR) spectral range are extensively applied in a variety of fields. These include, but are not limited to, nonlinear optics researches [8], soliton dynamics studies [9], hyperspectral LiDAR [10], and optical coherence tomography (OCT) [11, 12]. Recent years have witnessed urgent requirements for SC flatness, visible spectral coverage, and high power density across multiple domains. A representative case documented in ref. [13] demonstrated that power uniformity within the 400–800 nm visible band critically governs spectral reflectance inversion accuracy in hyperspectral LiDAR imaging. Applications imposing such rigorous specifications encompass optical tweezers [14, 15], fluorescence effect studies [16], sensing [17], light detection and ranging (LiDAR) [18]. Consequently, enhancing flatness, extending short‐wavelength boundaries, and improving visible‐band power density have emerged as prominent research frontiers. SC generation in picosecond‐pumped PCF is initially dominated by modulation instability (MI), with subsequent spectral broadening driven by synergistic effects including soliton fission, soliton self‐frequency shift (SSFS), stimulated Raman scattering (SRS), dispersive wave (DW) generation, and four‐wave mixing (FWM). To extend visible spectral coverage and enhance visible‐band power for improved flatness, strategies which exploit above nonlinear processes have been developed. Notable implementations include ytterbium‐doped fiber MOPA‐pumped cascaded dispersion‐decreasing PCFs, through which Travers et al. demonstrated visible to NIR SC spanning from 440 to 1890 nm (10‐dB bandwidth) in 2005 [19]. Zhang et al. subsequently achieved 370–2400 nm spectral coverage at 20‐dB bandwidth with two‐orders‐of‐magnitude power enhancement in 2020 [20]. 314.7 W output power with short‐wavelength extension to 390 nm (16‐dB bandwidth) has also been attained through long‐tapered PCF pumping techniques [21]. These approaches universally optimize PCF dispersion characteristics to shift the SC short‐wavelength boundary. Despite current SC average powers reaching hundreds of watts and spectral coverage extending into the visible range, substantial spectral power variations (> 10 dB) render the optical power across the region near the short‐wavelength edge insufficient for practical applications [20, 21, 22]. Consequently, spectral filtering becomes imperative for applications demanding high flatness. However, the requisite customized PCF fabrication, postprocessing (tapering and fusion splicing between PCFs), and filtering operations introduce significant manufacturing complexity, elevated costs, and compromised system reliability or stability. These constraints exacerbate thermal management challenges and fundamentally limit further SC power scaling. Consequently, generating ultraflat SC covering the visible to NIR spectrum has become an urgent research priority. Recent researches have shown significant progress. Explicitly, in 2024, Zhang et al. achieved 1100–2000 nm SC coverage with 7‐dB bandwidth by utilizing a 1063 nm pulsed laser to pump a 500‐m long phosphorus‐doped fiber [23]. In the same year, Jiang et al. implemented dual‐wavelength pumping of 100‐m SMF‐28e telecom fiber and generated SC spanning from 738 to 2200 nm at 6.5‐dB bandwidth [24]. This approach relies on ultralong fiber transmission to accumulate enough nonlinearity for SC flatness optimization which compromises the system compactness. In 2025, Yu et al. pumped a 27.55‐m long‐tapered PCF with a 1064 nm pulsed laser and obtained ultra‐flat SC with 3‐dB bandwidth spanning from 743 to 1988 nm [25]. Concurrently, Wang et al. engineered a specially structured PCF integrated with a long‐period fiber grating (LPFG) for spectral filtering of residual pump light. Then, by pumping 10‐m long PCF with a 1064 nm pulsed laser, they realized a SC spectral span of 492–2010 nm (3‐dB bandwidth) which also equals to 2.03 octaves [26]. This breakthrough represents a substantial advancement in ultra‐flat SC technology, extending coverage across most of the visible wavelengths.

Unlike conventional approaches, our proposed method employs chirp‐tunable high‐peak‐power pulses to pump low‐dispersion, highly nonlinear PCF, enhancing the FWM effect to rectify spectral imbalance. Generally, this imbalance occurs because the SRS, SSFS, and cascaded Raman peaks which all intensify the long‐wavelength spectral components relative to the blueshifted short wavelengths [24]. Crucially, although increasing the pulse chirp can enhance the visible light above 600 nm, excessive chirp degrades SC flatness. To overcome this limitation, we boost the pulse peak power to enhance the FWM‐driven DW generation below 600 nm and eventually extend the short‐wavelength boundary [27]. This mechanism efficiently transfers energy of spectral components (600–1000 nm) to shorter wavelengths (400–600 nm) whereas simultaneously suppress the continuously increase of spectral intensity above 600 nm induce by the gradually decreased pulse chirp.

By balancing PCF dispersion, chirp, and injected pulse peak power, we generate 4.35‐W ultraflat SC which spans from 414 to 1860 nm (3‐dB bandwidth). The 2.12‐octave SC covers almost all the visible band. To our knowledge, this represents the broadest octave‐spanning, visible‐to‐NIR, ultraflat SC source ever reported. Moreover, our method exhibits universal applicability. Explicitly, by optimizing the chirp and peak power of the injected pulse can effectively enhance the flatness of SC generated under any kind of PCF. The system stability, reliability, and compactness are also greatly improved by eliminating PCF postprocessing and spectral filtering setup. Hyperspectral LiDAR differs from conventional LiDAR systems, which rely on high coherence of the optical sources. Instead, it requires an SC which can provide broad, continuous spectral coverage, and high uniformity. Because these spectral characteristics are essential for improving the accuracy of spectral reflectance inversion, a critical process for material identification and analysis. With the advancement of SC generation techniques, broadband SCs have become a primary optical source solution for hyperspectral LiDAR. Especially, a visible‐band ultraflat SC source is an ideal candidate [26, 28, 29].

## 2 Simulation of SC Generation

### 2.1 Numerical Modeling of SC Generation in PCF Pumped by Chirped Pulse

To investigate the underlying mechanisms of visible‐enhanced ultraflat SC generation pumped by chirped pulse, we model the propagation of pulse in the PCF by adopting the generalized nonlinear Schrödinger equation (GNLSE) [30, 31].

(1) $$\frac{\partial A}{\partial z}+\frac{\alpha }{2}A-i\sum_{k\geq 2}\frac{i^{k}\beta_{k}}{k!}\frac{\partial^{k}A}{\partial t^{k}}=i\gamma \left(1+\frac{i}{\omega_{0}}\frac{\partial }{\partial t}\right)\times \left[A\left(z,t\right)\int_{-\infty }^{t}R\left(t^{\prime }\right){\left|A\left(z,t-t^{\prime }\right)\right|}^{2}dt^{\prime }\right],$$

here *A* = *A*(*z*, *t*) denotes the slowly varying amplitude and *α* represents the linear loss of the PCF which is neglected in our simulations. *β*_*k*_ is the kth‐order Taylor expansion term at the central frequency*ω*_0_. *γ* designates the nonlinear coefficient.

It is well‐established that increasing the pump pulse chirp can enhance the visible spectral components in SC generation [32], potentially elevating their intensity beyond that of the long‐wavelength bands. We initiate the numerical simulations by using 1030‐nm pulses with 14‐nm full‐width‐at‐half‐maximum (FWHM) spectral width and FWHM pulse duration of 3 ps. These pulses are propagated through single‐mode fibers of varied lengths (50, 100, 130, and 200 m) to induce different amount of chirp through temporal broadening processes, amplified, and subsequently injected into highly nonlinear PCF for SC generation. We assume a Gaussian pulse profile for the seed laser, deriving an initial chirp parameter *C* of 24 via the following expression:

(2) $$\Delta \omega ={\left(1+C^{2}\right)}^{1/2}/T_{0},$$

where *T*_0_ is the pulse half‐width at 1/e intensity, Δ*ω* denotes the spectral half‐width and *C* represents the chirp parameter. We employ the Gaussian pulse propagation model detailedly described in ref. [30].

(3) $$A\left(t\right)=\sqrt{P_{0}}\exp\left[-\frac{\left(1+iC_{1}\right)}{2}\frac{T^{2}}{T_{1}^{2}}\right],$$

where

(4) $$T_{1}=T_{0}\sqrt{{\left(1+\frac{C\beta_{2}L}{T_{0}^{2}}\right)}^{2}+{\left(\frac{\beta_{2}L}{T_{0}^{2}}\right)}^{2},}$$

(5) $$C_{1}=C+\left(1+C^{2}\right)\frac{\beta_{2}L}{T_{0}^{2}}.$$

*P*_0_ denotes the peak power of the input pulse, *β*_2_ = 2.22 × 10^−26^ s^2^/m and *L* represent GVD parameter and the transmission length of single‐mode fiber. The calculated temporal broadening and chirp parameter corresponding to different lengths of single‐mode fiber are presented in Figure 1a.

**FIGURE 1:**
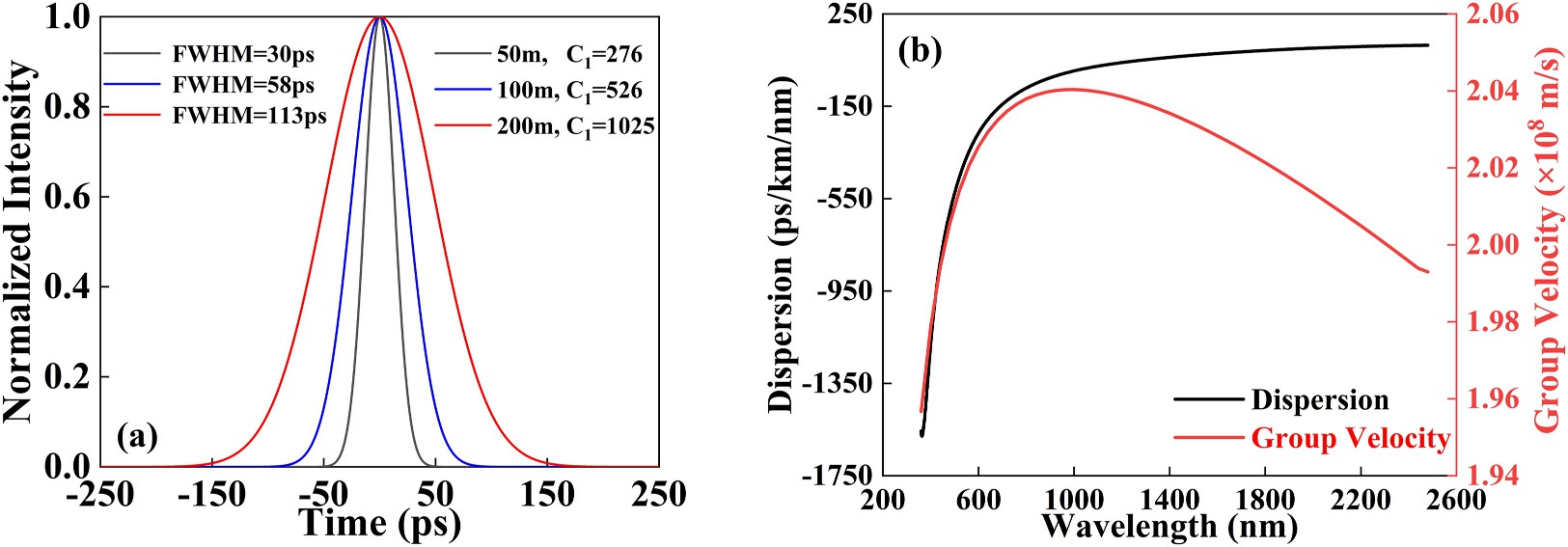
(a) Temporal broadening and chirp parameter calculation results and (b) the dispersion curve and group velocity of the highly nonlinear PCF.

With repetition rate of the seed laser fixed at 31 MHz, the amplifier delivers the maximum output power of 14 W. The calculated pulse peak powers corresponding to different single‐mode fiber lengths are tabulated in Table 1. The dispersion curve of the 5‐m long highly nonlinear PCF is shown in Figure 1b which exhibits a zero‐dispersion wavelength (ZDW) of around 986 nm. The dispersion coefficients are further derived from the Taylor expansion of the highly nonlinear PCF’s propagation constant at 1030 nm. Specifically, *β*_2_ = −5.72797 × 10^−27^ s^2^/m, *β*_3_ = 7.58044 × 10^−41^ s^3^/m, *β*_4_ = −5.20068 × 10^−56^ s^4^/m, *β*_5_ = 4.80835 × 10^−71^ s^5^/m, *β*_6_ = −5.23153 × 10^−86^ s^6^/m, *β*_7_ = 4.33026 × 10^−101^ s^7^/m, *β*_8_ = −2.02499 × 10^−116^ s^8^/m, *β*_9_ = 4.72881 × 10^−132^ s^9^/m, and *β*_10_ = −4.19943 × 10^−148^ s^10^/m. The nonlinear coefficient *γ* of the highly nonlinear fiber is 18 W^−1^ km^−1^. According to the split‐step Fourier method and Nyquist sampling theorem, the temporal step size *dt* and spatial step size *h* are configured as 0.4 fs and 5 μm. Exact modeling the propagation of hundred picosecond pulse requires that *dt* = 0.4 fs (temporal) and *h* = 5 μm (spatial) according to the split‐step Fourier and Nyquist sampling theorem. With the temporal points *N*_*T*_ selected as 2^22^, we deploy GPU acceleration, consolidated computation, and adaptive sampling to resolve the temporal and spectral dynamics during SC generation.

**TABLE 1:** Transmission fiber lengths for seed pulse broadening and the corresponding pulse peak power after amplification.

Transmission fiber length (m)	Pulse peak power (kW)
50	15.1
100	7.8
200	4.0

### 2.2 Results and Discussion

Figure 2 exhibits the calculated SC spectra under different input chirp at the propagation end of 5‐m long highly nonlinear PCF. For the case of C_1_ = 1025, it can be clearly seen that the intensity of the spectral components on the long wavelength side is large than that of the visible band. Meanwhile, the short wavelength boundary locates at 466 nm. In contrast, when the input chirp parameter C_1_ is decreased to 526, evidently, the spectral intensity of the visible band increases. The short‐wavelength boundary reaches 434 nm. When we further decrease the chirp parameter C_1_ to 276, the edge on the short wavelength side is extended to 425 nm resulted from the enhanced pulse peak power. The detailed temporal and spectral evolution within the 5‐m long PCF is illustrated in Figure 3 for chirp values C_1_ = 526, 1025, and 276. By varying C_1_, distinct SC dynamics can be observed due to the interactions of chirp and pulse peak power, manifesting as divergent DW emission positions and pulse fission patterns. Comparative simulations indicate that the coordinate effects of input pulse chirp, peak power, and dispersion of the highly nonlinear PCF can significantly improve the SC flatness, enhance the spectral intensity of visible band and extend the short‐wavelength edge.

**FIGURE 2:**
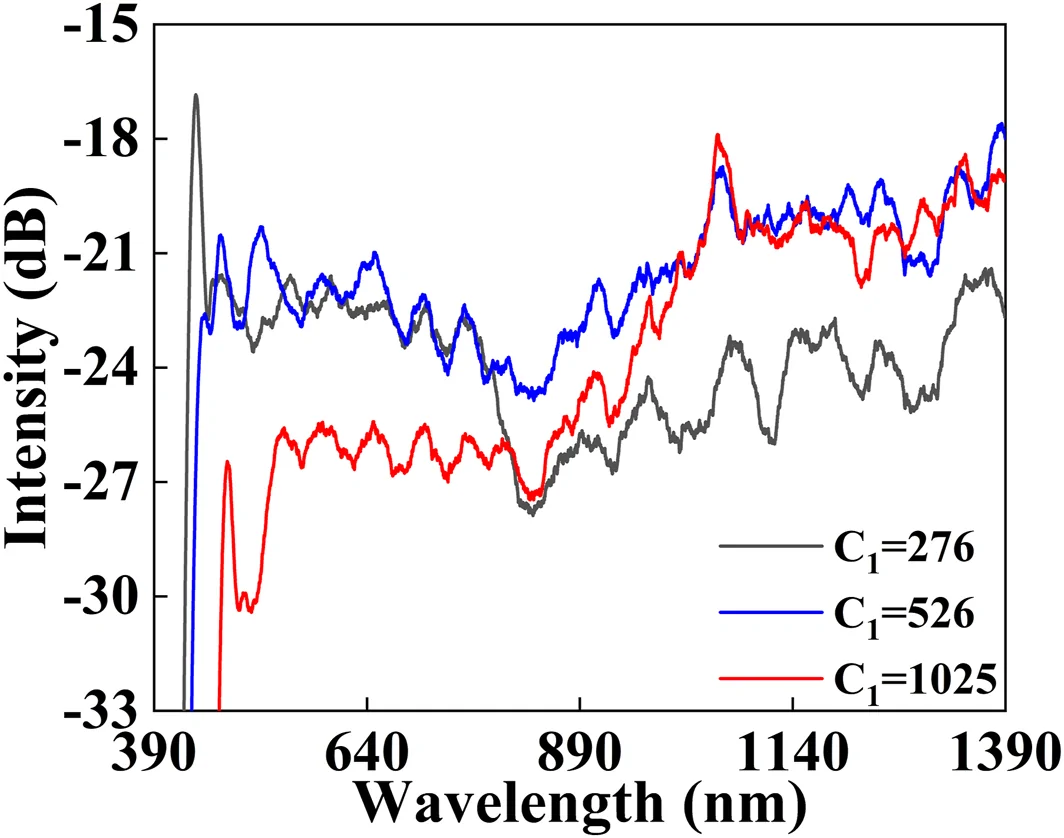
Simulated SC spectra for different chirp values, C_1_ = 276, 526, and 1025 at the end of 5‐m highly nonlinear PCF.

**FIGURE 3:**
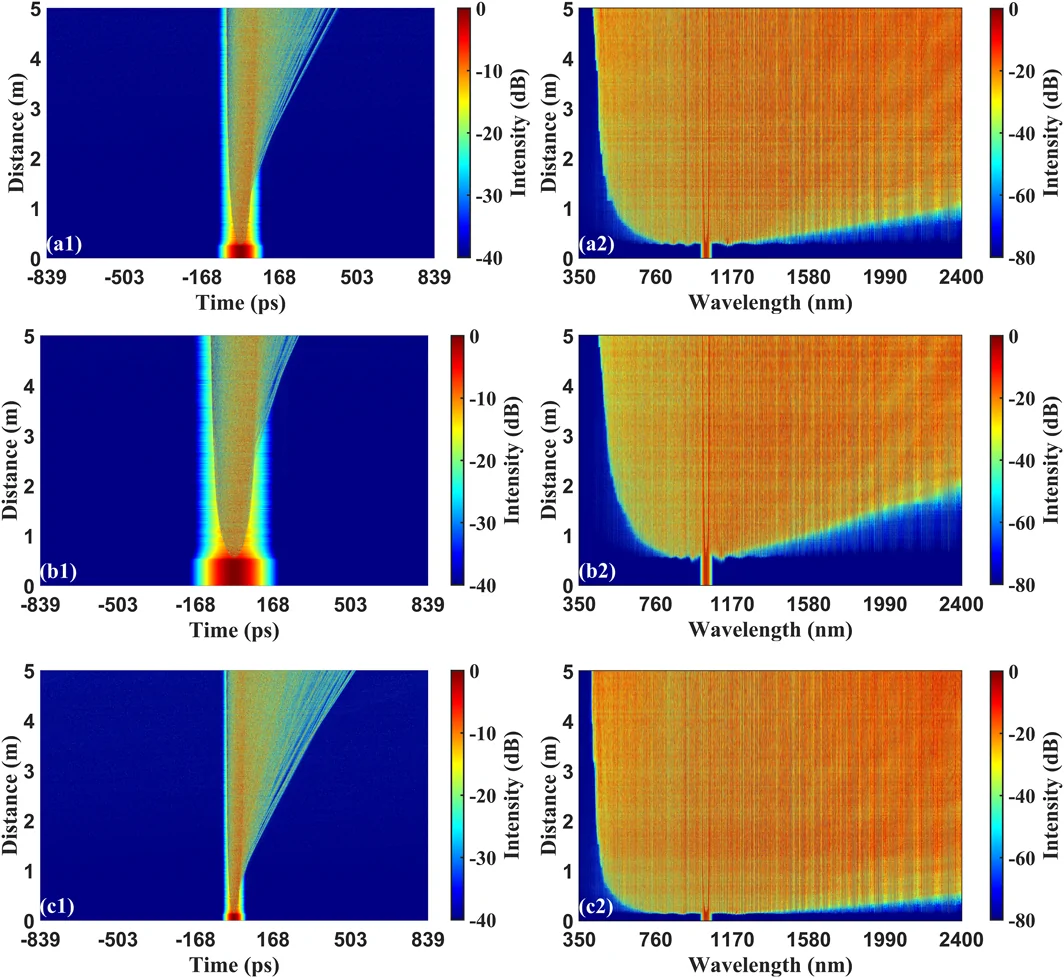
Simulated SC evolution process for chirp values: (a) C_1_ = 526, (b) C_1_ = 1025, and (c) C_1_ = 276.

## 3 Experimental Verification

### 3.1 Experimental Setup

As shown in Figure 4, we construct an all‐fiber SC generation system based on chirp‐tunable pump pulses. The pump pulses of highly nonlinear PCF are delivered from a MOPA system. Within the MOPA configuration, the seed light comes from a 1030 nm, 31‐MHz repetition rate mode locked fiber laser. The seed pulse acquires varying chirp magnitude through propagation in SMF‐28e fibers with different lengths. In the preamplification stage, an 80‐cm long single‐mode ytterbium‐doped fiber (YDF and Yb401) is used to elevate average power of the seed laser from 20 to 200 mW. The main amplifier constructed by using 170‐cm long 10/125 μm YDF further increases the average power to 14.18 W. Eventually, the amplified pulse is directly injected into 5‐m long highly nonlinear PCF to produce SC. An endcap is adopted to protect the output interface and prevent back‐reflection‐induced power instability. The SC are collected by using two optical spectrum analyzers: an ANDO AQ6315 OSA (350–1750 nm spectral range) and a Yokogawa AQ6375B OSA (1200–2400 nm measurement range) with an FELH1200 filter.

**FIGURE 4:**
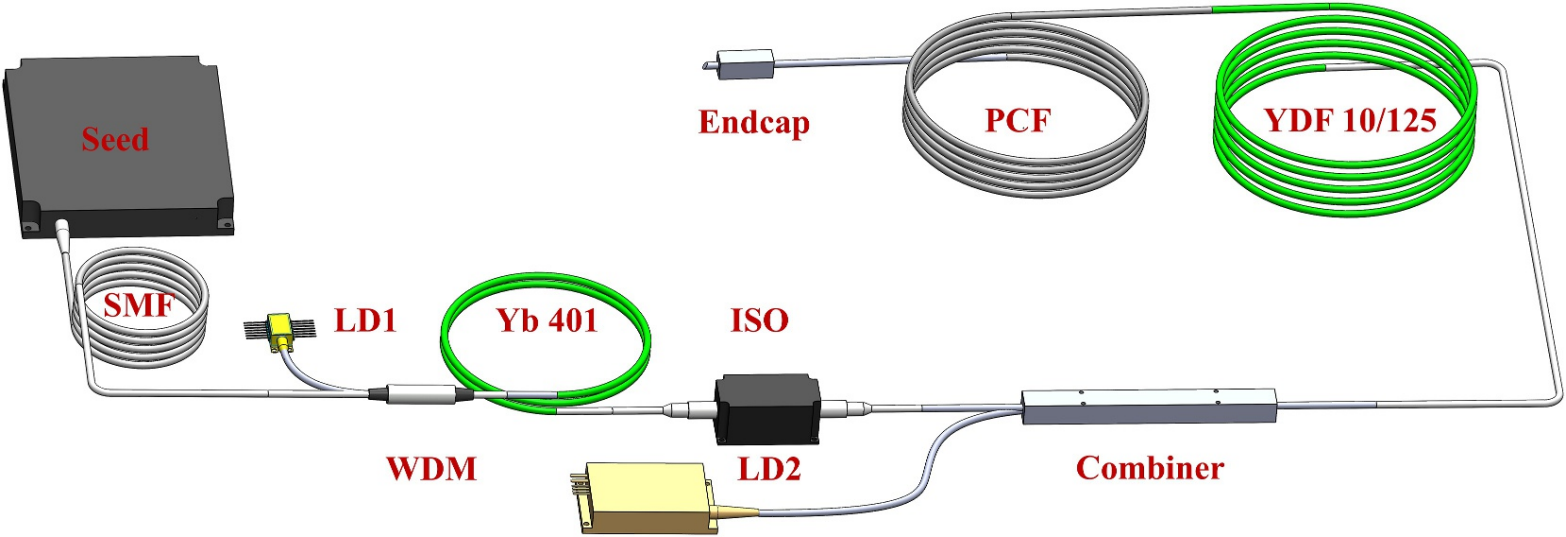
The schematic diagram of all fiber‐based ultraflat visible SC generation system.

### 3.2 Results and Discussion

Figure 5 shows the measured autocorrelation traces of the seed pulse and broadened pulses corresponding to different SMF‐28e fiber lengths. Take 200‐m SMF‐28e fiber, for example, the initial 3‐ps seed pulse is broadened to 88 ps. After two‐stage amplification, the output power reaches 14.18 W. The amplified signal power versus the pump power and the optical spectrum at the maximum output power are presented in Figure 6.

**FIGURE 5:**
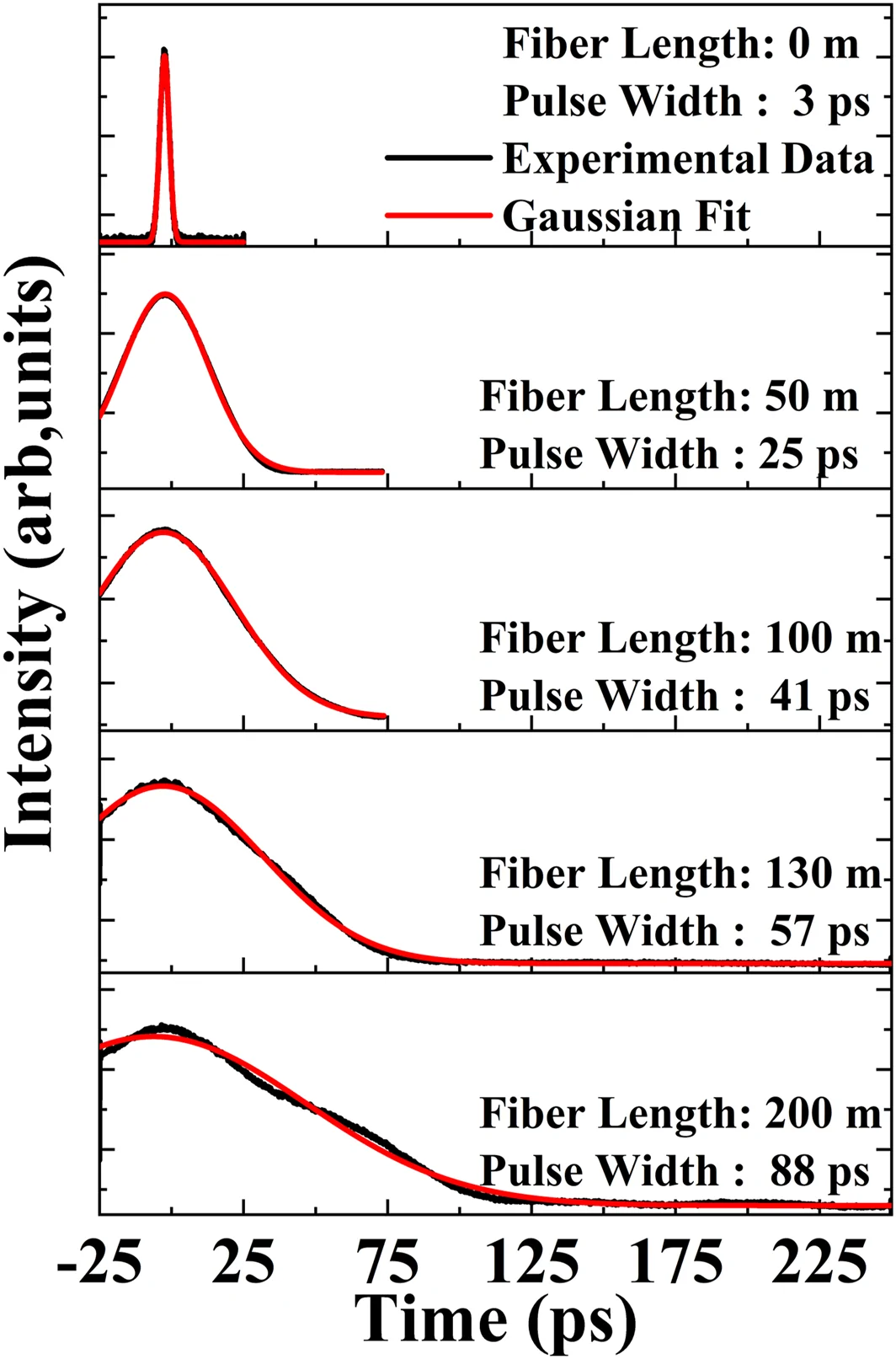
Autocorrelation traces of the seed pulse and broadened pulses corresponding to different SMF‐28e fiber lengths. Gaussian fitting of the measured traces are also shown in the figure.

**FIGURE 6:**
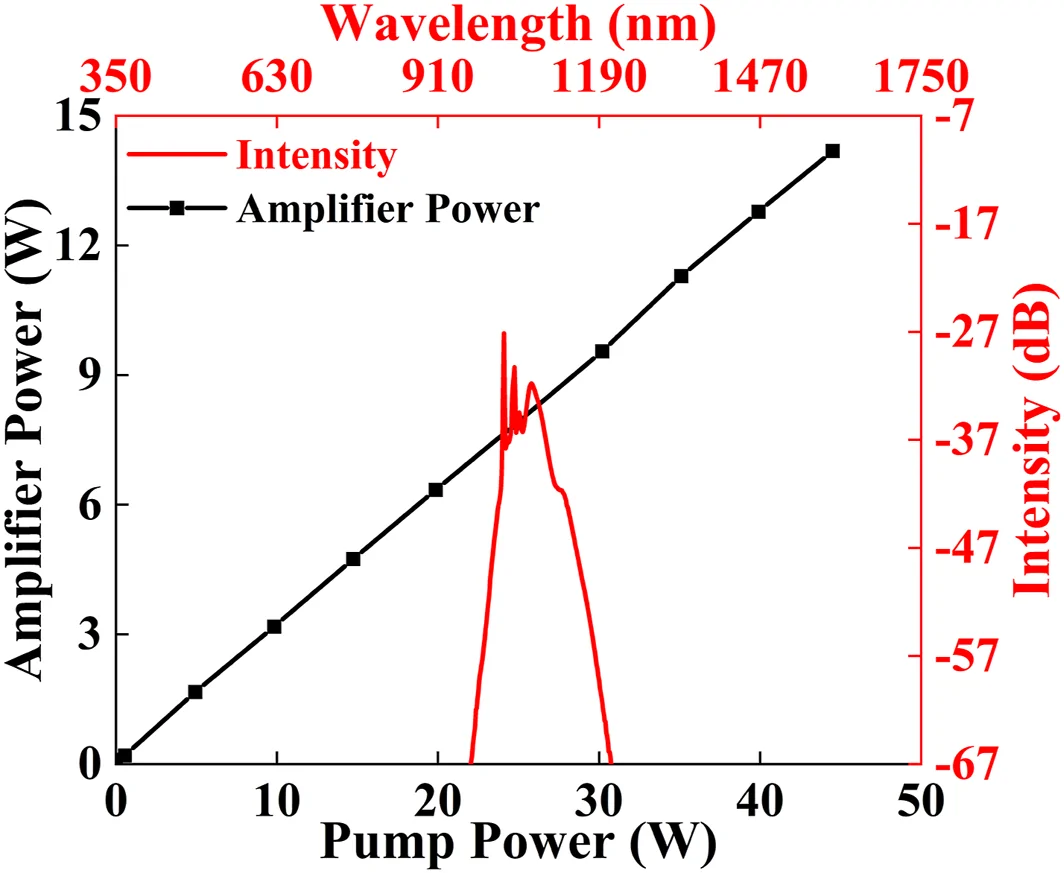
Variation of the amplified signal power with the pump power as well as the optical spectrum at the maximum output power.

The measured SC spectra are presented in Figure 7. The lengths of the SMF‐28e fiber for pulse stretching are 100, 130, and 200 m. As shown in Figure 7, SC generated by injecting pulses prechirped through 100‐m SMF‐28e fiber exhibits enhanced visible light. The intensity of short‐wavelength, especially 600–930 nm wavelength band clearly exceeds that of the long‐wavelength side. Conversely, for the case of 200‐m SMF‐28e, the intensity of the short‐wavelength side drops below that of the redshifted long‐wavelength side resulted from the excessive chirp. We further experimentally validate that 130‐m long SMF‐28e can introduce optimal chirp and SC with a flat spectral profile is eventually obtained. Meanwhile, this ultra‐flat SC exhibits a short‐wavelength boundary of 525 nm (3‐dB bandwidth). Although a proper pulse chirp can significantly reduce the intensity difference between short‐ and long‐wavelength sides to below 3 dB, the enhancement of visible band primarily occurs in the wavelength region of 600–1000 nm and the spectral boundary extension is still insufficient for the purpose of an ultraflat visible to NIR SC.

**FIGURE 7:**
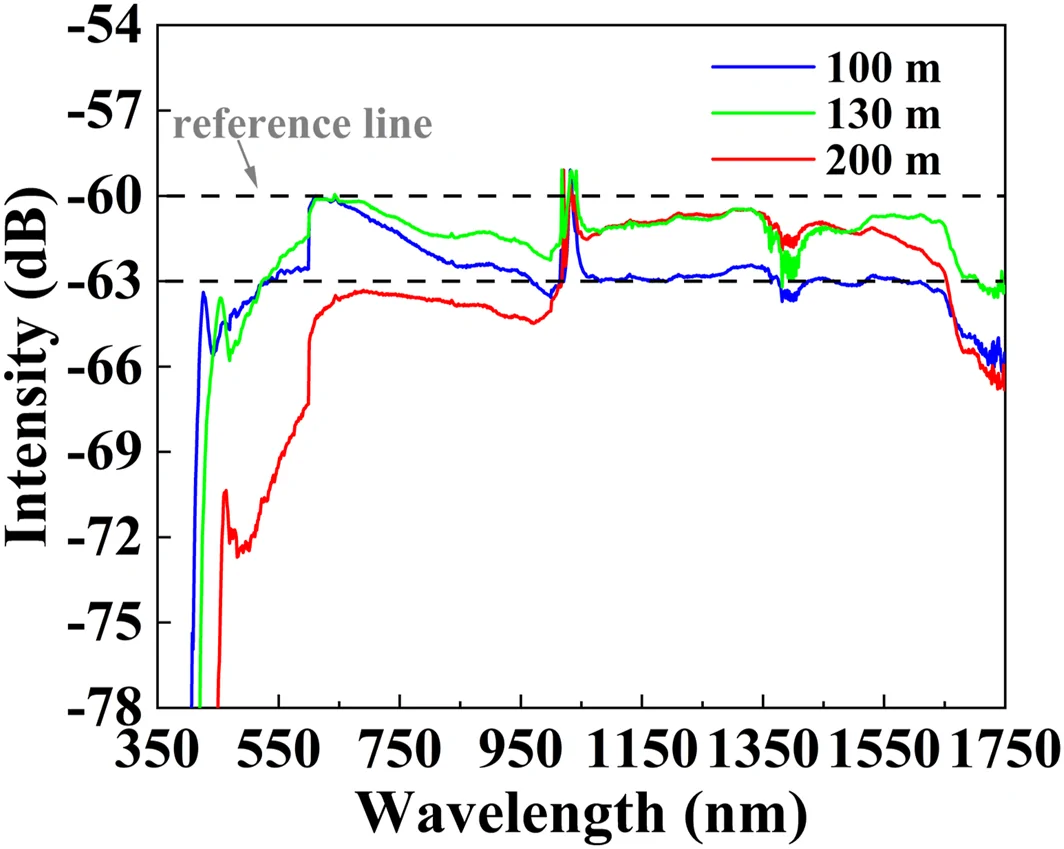
Experimental validation of input pulse chirp tailored SC. SC spectra relating to 100‐, 130‐, and 200‐m SMF‐28e fiber are shown. The dashed lines indicate 3‐dB bandwidth.

As illustrated in Figure 3, under the optimal chirp parameter of C_1_ = 276, the pulse‐splitting dynamics are controlled to enable repeated temporal overlap and collision between soliton and the DW within the finite‐length highly nonlinear PCF. This sustained interaction drives efficient FWM and enhances the generation of short‐wavelength components. Although adjusting the chirp parameter C_1_ from 1025 to 526 initiates this process, the corresponding pulse peak power of 7.8 kW remains insufficient to maintain efficient FWM. In the experiment, by further decreasing the chirp value to 276 through reducing the prepulse broadening amount, the peak power is increased to 15.1 kW. The higher pulse peak power can effectively sustain the FWM process, strengthen the 400–600 nm band and enable continued extension of the short‐wavelength edge. For comparison, a numerical simulation was performed to verify this point and the result is shown in Figure 8. Explicitly, fixing the chirp parameter at C_1_ = 526, a distinct blue shift of the short‐wavelength edge is observed when increase the pulse peak power from 7.8 to 15.1 kW. This confirms the critical role of pulse peak power.

**FIGURE 8:**
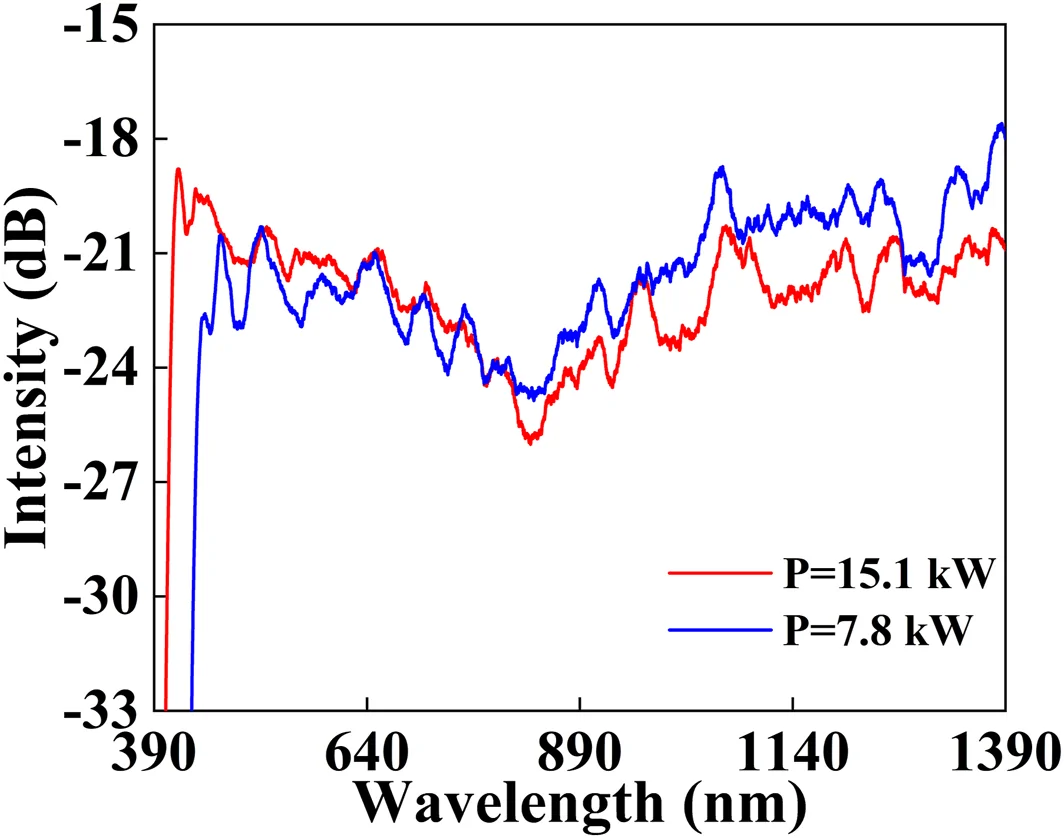
Simulated SC spectra under a fixed chirp parameter of C_1_ = 526 and different pulse peak powers of 15.1 kW (red line) and 7.8 kW (blue line).

As is also experimentally evidenced, we achieve the optimal balance among the PCF dispersion, input pulse chirp, and peak power by using 50‐m long SMF‐28e fiber to stretch the seed pulse. As shown in Figure 9, we generate 4.35‐W, ultraflat SC spanning from 414 to 1860 nm (3‐dB bandwidth). The intensity fluctuation in the NIR region of the SC spectrum shown in Figure 9a is attributed to limited sensitivity of the measurement setup (Yokogawa AQ6375B OSA) and power attenuation induced by the integrating sphere used for SC collection. Nonetheless, the intensity variation remains within the 3‐dB range and does not affects the evaluation of 3‐dB spectral width and SC spectral coverage. Figure 9b gives the variation of the SC power with the pump power, which reveals a 31% optical‐to‐optical conversion efficiency at the maximum output power.

**FIGURE 9:**
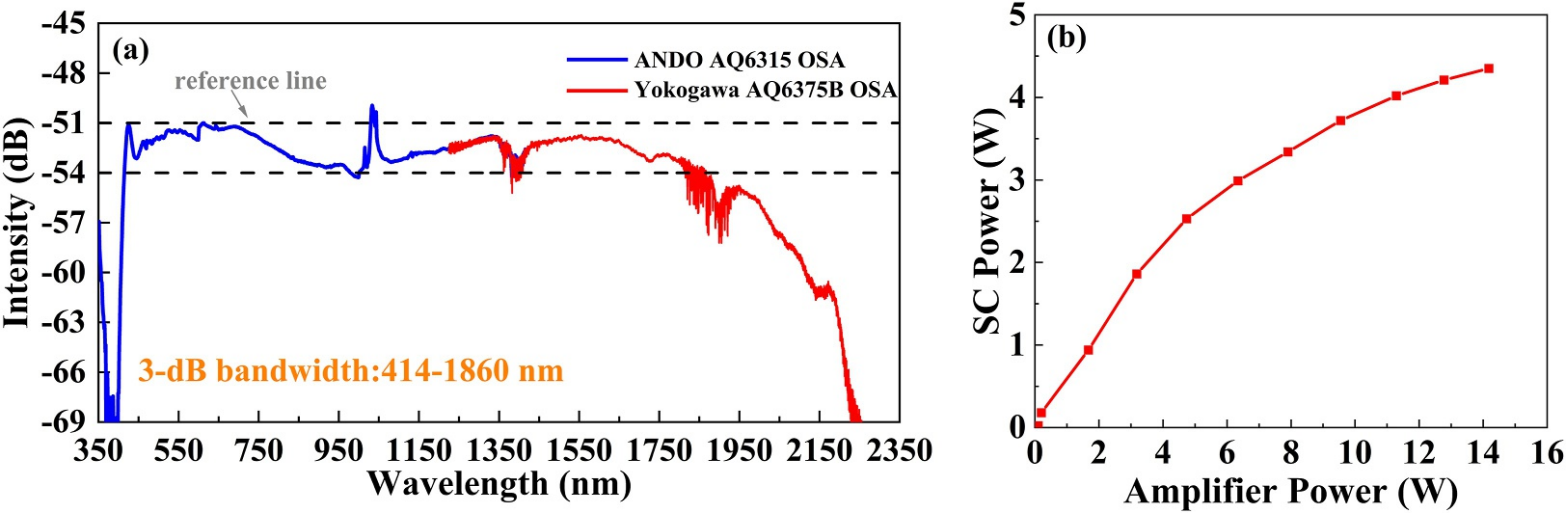
(a) Visible‐enhanced ultra‐flat SC generation benefitting from the balance among PCF dispersion, input pulse chirp (C_1_ = 276), and pulse peak power (*P* = 15.1 kW). (b) Variation of the SC power with the pump power.

The spectral broadening toward shorter wavelengths is primarily driven by two mechanisms: DW radiation from red‐shifted solitons and FWM between the solitons and those DWs. The phase‐matching condition between solitons and DWs is given by refs. [33, 34]:

(6) $$\sum_{k\geq 2}\frac{\beta_{k}}{k!}{\left(\omega_{DW}-\omega_{s}\right)}^{k}=\frac{\gamma P_{s}}{2},$$

where *ω*_s_ is the soliton center frequency, *β*_*k*_ (*ω*_s_) denotes the *k*th‐order derivative of the propagation constant *β* at *ω*_s_ (from Taylor expansion), *γ* is the nonlinear coefficient, and *P*_s_, the soliton peak power, is set to 15.1 kW. Substituting the parameters of the abovementioned highly nonlinear PCF into Equation (6), the phase‐matching relationship between solitons and DWs is obtained as shown in Figure 10. The radiated DW has a wavelength of approximately 400 nm, which would require a matched soliton wavelength of about 2400 nm. However, the long‐wavelength edge of the SC spectrum in Figure 9a is far from this value. The seed pulse, without any prechirp control (without prior broadening in SMF‐28e fiber), is directly amplified to a peak power of 15 kW and injected into the highly nonlinear PCF. The SC spectrum in Figure 11 shows a short‐wavelength edge of 460 nm and a spectral power variation > 6 dB, which differs significantly from the 414 nm. Therefore, the mechanism of soliton‐radiated DWs alone is insufficient for generating the ultraflat SC.

**FIGURE 10:**
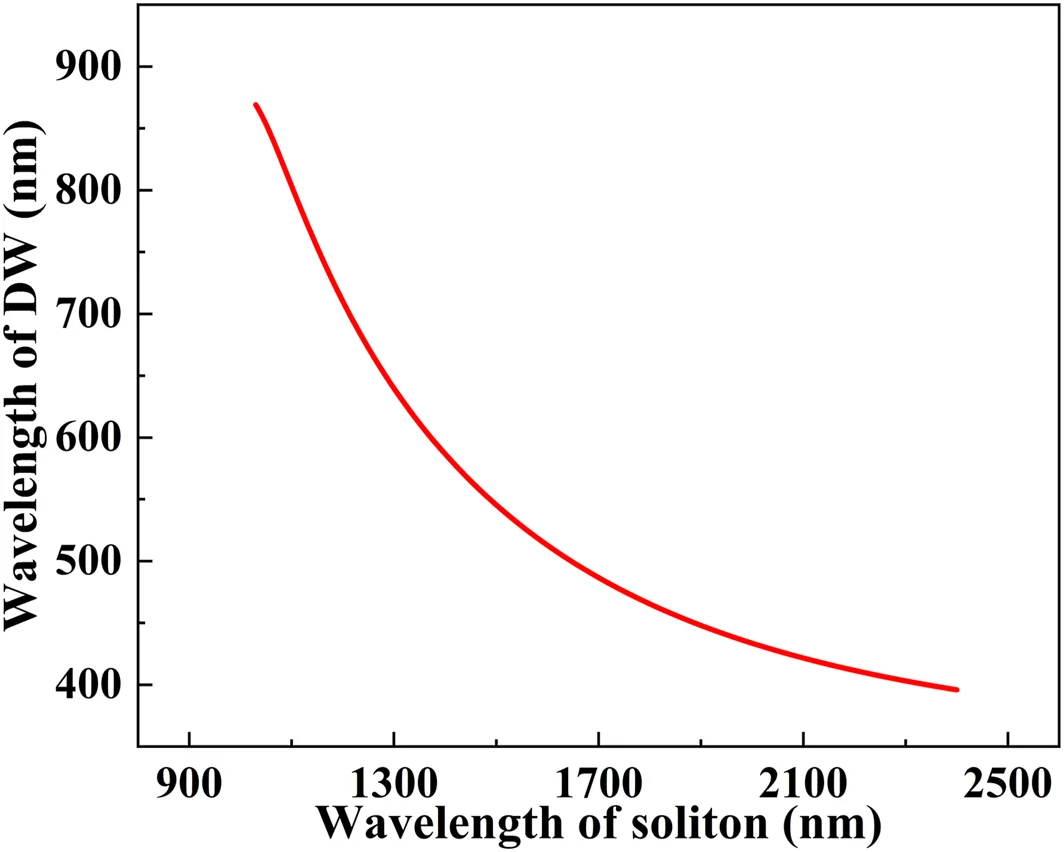
Phase matching relationship between solitons and DWs.

**FIGURE 11:**
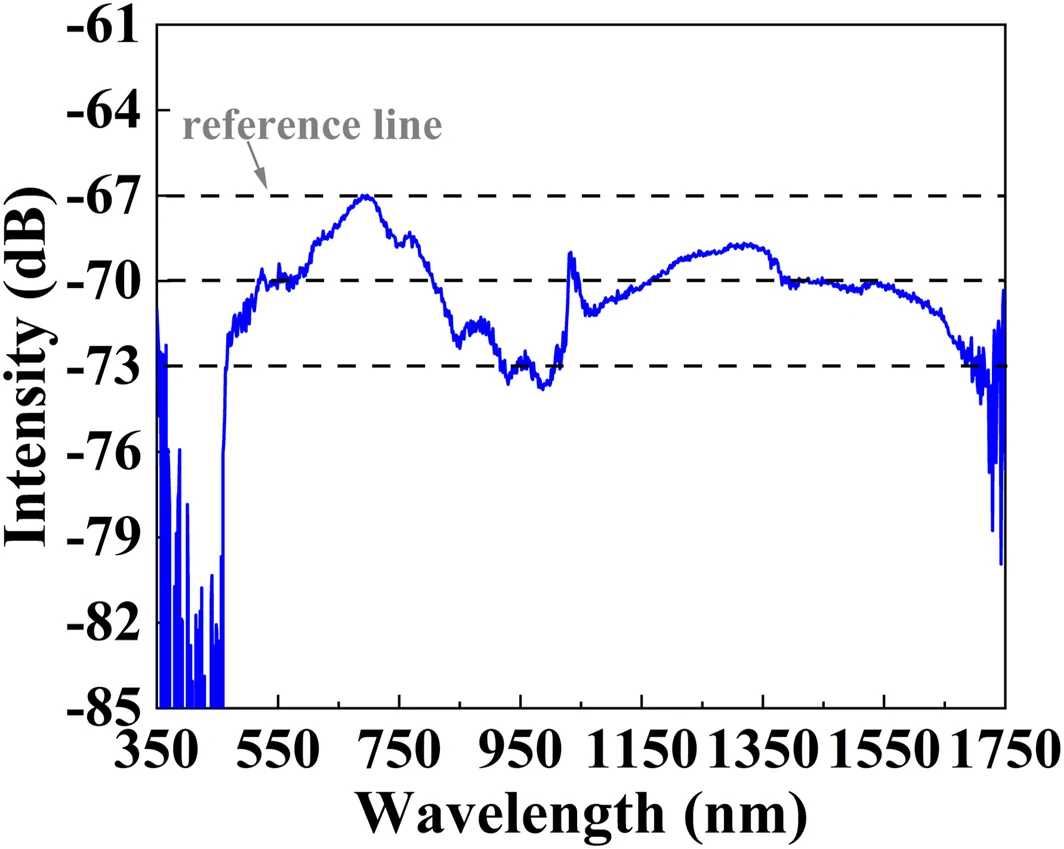
Experimentally measured SC spectrum. The seed pulse, without prior broadening in SMF‐28e fiber, is directly amplified to a peak power of 15 kW and then injected into the highly nonlinear PCF.

We numerically simulate the evolution of SC spectra when the pump pulse propagates along the highly nonlinear PCF under the experimental conditions present in Figure 9. As shown in Figure 12, under the given optimal chirp of C_1_ = 276 and peak power of 15.1 kW, we control the pulse‐splitting dynamics so that the temporal overlap and collision between soliton and the DW can be maintained within the finite‐length highly nonlinear PCF length. This long‐term interactions can support efficient FWM processes, which results to intensity strengthening of the visible band and short‐wavelength edge extending of the SC spectrum. Therefore, the strategy described above relies on chirp optimization to control the soliton‐DW temporal overlap and increasing the pulse peak power to sustain efficient FWM rather than on the mechanism of DW radiation from solitons.

**FIGURE 12:**
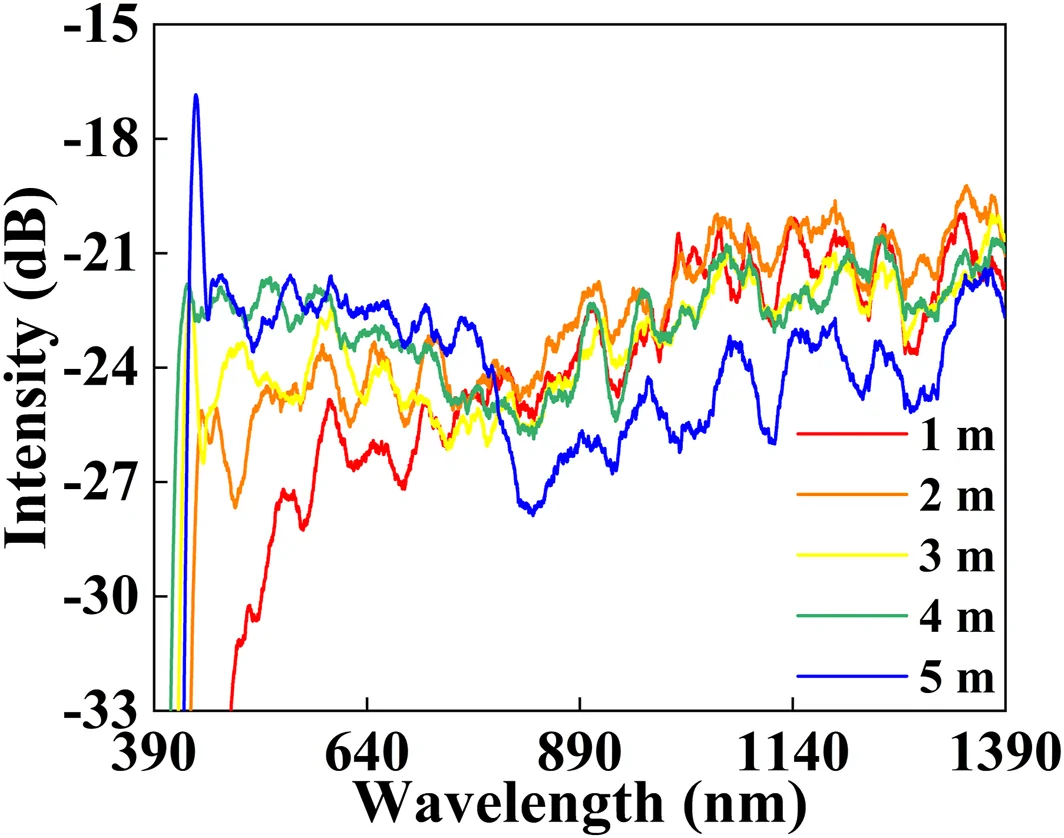
Numerically simulated SC spectra relating to propagation distances of 1, 2, 3, 4, and 5 m in the HN‐PCF. Parameters: C_1_ = 276 and *P* = 15.1 kW.

## 4 Conclusion

In summary, due to the SRS, SSFS, and cascaded Raman peaks, for visible to NIR SC, the intensity of long‐wavelength spectral components is usually higher than that of the short wavelengths [24]. Through numerical modeling and experimental validation, we propose an improving methodology of SC flatness by optimizing the combined effects of highly nonlinear PCF dispersion, input pulse chirp, and peak power. Specifically, when the optimized state of the combination of above three parameters are arrived, the FWM effect can be effectively enhanced to elevate the spectral intensity of visible band. As a result, the SC flatness is significantly improved and the short‐wavelength edge is also extended. Based on this approach, we experimentally generate 4.35‐W, ultraflat SC spanning from 414 to 1860 nm (3‐dB bandwidth and 2.12 octave), which nearly encompasses the entire visible band. As benchmarked in Table 2, we achieve the broadest visible to NIR SC source with the maximum visible spectral coverage (3 dB bandwidth).

**TABLE 2:** Summary of ultraflat NIR and visible to NIR SC sources.

Year	Spectrum (without considering the residual pump)	Power (W)	Optical octave	Pump wavelength (nm)	Fiber	Length (m)	Reference
2024	1100‐2000@7dB	0.138	0.82	1063	Phosphorus‐doped fiber	500	[23]
2024	1600‐2200@5dB	1.82	0.38	1560	GeO_2_ fiber	0.15	[35]
2024	738‐2200@6.5dB	—	1.50	1030&1550	SMF 28e	100	[24]
2025	743‐1988@3dB	5.1	1.34	1064	Tapered‐PCF	27.55	[25]
2025	492‐2010@3dB	0.815	2.02	1064	PCF	10	[26]
2025	414‐1860@3dB	4.35	2.12	1030	PCF	5	Our work

Compared with the existing techniques, our method bears the virtue of universal applicability for the spectral flatness enhancement of SC generated in arbitrary kind of highly nonlinear PCF. Moreover, we generate watt‐level (> 4 W) ultra‐flat SC with unprecedented visible band coverage by using only 5‐m long highly nonlinear PCF.

## Author Contributions

**Yashuai Guo:** conceptualization, methodology, software, investigation, validation, writing. **Xiaohong Hu:** sofware, review. **Ting Zhang:** investigation. **Ran Pan:** resources, data curation. **Wei Zhang:** methodology, investigation, project administration. **Yishan Wang:** funding acquisition, supervision. **Wei Zhao:** visualization.

## Funding

This study was supported by National Natural Science Foundation of China (Grant 52327808); Western Young Scholars Program of CAS (XAB2020YN11); and Key Research and Development Program of Shaanxi Province 2025SF‐YBXM‐519.

## Conflicts of Interest

The authors declare no conflicts of interest.

## Data Availability

Data underlying the results presented in this paper are not publicly available at this time but may be obtained from the authors upon reasonable request.
